# Imaging and proteomics toolkits for studying organelle contact sites

**DOI:** 10.3389/fcell.2024.1466915

**Published:** 2024-09-24

**Authors:** Rico Gamuyao, Chi-Lun Chang

**Affiliations:** Department of Cell and Molecular Biology, St. Jude Children’s Research Hospital, Memphis, TN, United States

**Keywords:** organelle contact sites, electron microscopy, light microscopy, bimolecular complementation, split fluorescent protein, splitFAST, proximity labeling, split-TurboID

## Abstract

Organelle contact sites are regions where two heterologous membranes are juxtaposed by molecular tethering complexes. These contact sites are important in inter-organelle communication and cellular functional integration. However, visualizing these minute foci and identifying contact site proteomes have been challenging. In recent years, fluorescence-based methods have been developed to visualize the dynamic physical interaction of organelles while proximity labeling approaches facilitate the profiling of proteomes at contact sites. In this review, we explain the design principle for these contact site reporters: a dual-organelle interaction mechanism based on how endogenous tethers and/or tethering complexes localize to contact sites. We classify the contact site reporters into three categories: (i) single-protein systems, (ii) two-component systems with activated reporter signal upon organelle proximity, and (iii) reporters for contact site proteomes. We also highlight advanced imaging analysis with high temporal-spatial resolution and the use of machine-learning algorithms for detecting contact sites.

## 1 Introduction

Eukaryotic cells contain various organelles with specialized functional roles. While they are spatially segregated and have long been considered independent entities, they communicate and work together to maintain cellular homeostasis and function. One significant platform of inter-organelle communication is through contact sites, where heterologous organelles juxtaposed within nanometers to facilitate inter-organelle signaling, lipid transport, metabolic channeling, and regulation of organelle morphology and biogenesis (Prinz et al., 2020; Voeltz et al., 2024). Defects in organelle contact sites have been associated with neurological diseases (Kim et al., 2022), diabetes (Wang et al., 2022; Liu et al., 2023), obesity (Arruda et al., 2014; Parlakgul et al., 2022), and cancer (Kerkhofs et al., 2017; Zhang et al., 2019; Shuwen et al., 2023), emphasizing the physiological relevance of organelle interaction.

Organelle contact sites were first observed in the 1950s via electron microscopy (EM), demonstrating a physical association between the endoplasmic reticulum (ER) and mitochondria and between the ER and plasma membrane (PM) (Bernhard et al., 1952; Fawcett, 1955; Bernhard and Rouiller, 1956; Porter and Palade, 1957; Copeland and Dalton, 1959). Nonetheless, the significance of organelle interaction remained elusive for decades due to the lack of functional data. It was only in the 1990s that mitochondria-associated ER membrane fractions were demonstrated to have a functional role in importing phosphatidylserine into the mitochondria (Vance, 1990; Vance, 1991). In yeast, ER-mitochondria contact sites, or ER-mitochondria encounter structure (ERMES) complex, are maintained by Mdm10, Mdm12, Mdm34, and Mmm1 (Kornmann et al., 2009). The ERMES is vital for the efficient transfer of phospholipids between these organelles (Kornmann et al., 2009; Voss et al., 2012; AhYoung et al., 2015; Kawano et al., 2018). These contact sites also provide spatial regulation for efficient calcium transfer between the ER and mitochondria (Rizzuto et al., 1993; Rizzuto et al., 1998; Csordas et al., 2010). Over the past two decades, organelle contact sites became a burgeoning field, leading to mechanistic and functional insights into these minute subcellular foci between virtually all organelles. For example, lipid droplet (LD)-organelle interactions play pivotal roles in fatty acid metabolism. While LD-ER contact sites promote LD biogenesis (Robenek et al., 2006; Salo et al., 2016; Wang et al., 2016; Datta et al., 2019), mitochondria-LD contact sites are involved in both fatty acid oxidation (Sadh et al., 2017; Wang et al., 2021; Miner et al., 2023; Talari et al., 2023) and storage of excess lipids (Benador et al., 2018; Najt et al., 2023). Moreover, contact sites between peroxisomes and LDs promote fatty acid trafficking during lipolysis (Binns et al., 2006; Kong et al., 2020) and facilitate the removal of lipid peroxides (Chang et al., 2019). Due to the space limitation of this minireview, we could not cover all the relevant literature. Recent progress in the contact site field can be found in several other reviews (Eisenberg-Bord et al., 2016; Scorrano et al., 2019; Prinz et al., 2020; Voeltz et al., 2024).

Organelle contact sites are dynamically maintained and regulated via molecular tethering complexes that constitute dual-organelle interaction mechanisms which bridge two organelles in close proximity. Thus, visualizing the dynamism and identifying protein regulators of contact sites have always been the key and challenging issues to uncovering the functional significance of contact sites. While the field learned how endogenous tethers work, researchers have adapted the tethering principle to develop molecular tools to tackle these issues. In this minireview, we first discuss the technical challenges of studying organelle contact sites and then focus on how a variety of molecular tools can help resolve these issues and move this field forward.

## 2 Challenges in studying organelle contact sites

The most common challenge when studying contact sites is the detection of these sites. The intuitive, straightforward method to visualize organelle contact sites is through microscopy techniques. EM is considered the gold standard as it has a sufficient spatial resolution for the detection of contact sites ∼200 nm in size. However, EM-based approaches cannot capture the dynamic nature of organelle contact sites in living cells, as the samples must be fixed. Moreover, the time-consuming sample preparation limits the sampling size and throughput to acquire statistically meaningful data. Alternatively, light microscopy (LM) combined with colocalization analysis between fluorescently labeled organelles captures the dynamic nature of contact sites and overcomes the throughput issue in EM. In fact, multispectral imaging capable of simultaneous detection up to seven organelles provides systematic readouts of organelle interactomes in many cell types (Valm et al., 2017; Zimmermann et al., 2024). However, the spatial resolution of LM is insufficient for accurately measuring individual contact sites. In addition, the application of the LM-colocalization approach is often restricted to flat, adherent cell lines, hindering our understanding of contact site biology in physiologically relevant cellular systems. Super-resolution imaging techniques with lateral resolutions up to ∼20 nm appeared to be the solution for the LM-colocalization approach and have been applied to visualize many organelle contact sites (Shim et al., 2012; Raiborg et al., 2015; Bottanelli et al., 2016; Hua et al., 2017; Modi et al., 2019; Damenti et al., 2021). Nonetheless, the requirement of high signals during super-resolution imaging often leads to photobleaching and reduced temporal information. In addition, super-resolution microscopes are often only available in imaging facilities due to their high cost and maintenance.

Another difficulty of this field is the unbiased identification of protein components at contact sites during mechanistic interrogation. Traditional biochemical purification of contact site fractions from the whole cell lysate and subsequent proteomic analysis made inroads (Poston et al., 2013; Prasad et al., 2015), but it remains technically challenging to preserve organelle contact site integrity and minimize cross-contamination. Targeted approaches, such as immunoprecipitation of known contact site proteins, appeared to be a feasible method to identify new components. However, large-scale and unbiased identification remains difficult with this method. Proximity labeling approaches via targeting the ascorbate peroxidase (APEX/APEX2) (Rhee et al., 2013; Lam et al., 2015) or BioID engineered from bacterial biotin ligase (Roux et al., 2012) to one organelle have been applied to identify contact site components. As proximity labeling substantially improves the throughput, these approaches require additional filtering steps, either experimentally or analytically, to identify purported contact sites based on known protein localization and/or function (Cho et al., 2017; Hua et al., 2017; Hung et al., 2017; van Vliet et al., 2017; Cabukusta et al., 2020), hindering unbiased identification of new contact site proteome. In the following sections, we discuss recently developed molecular tools that will help tackle visualization challenges and unbiased proteome identification of organelle contact sites.

## 3 Design principle for organelle contact site reporters

**FIGURE 1:**
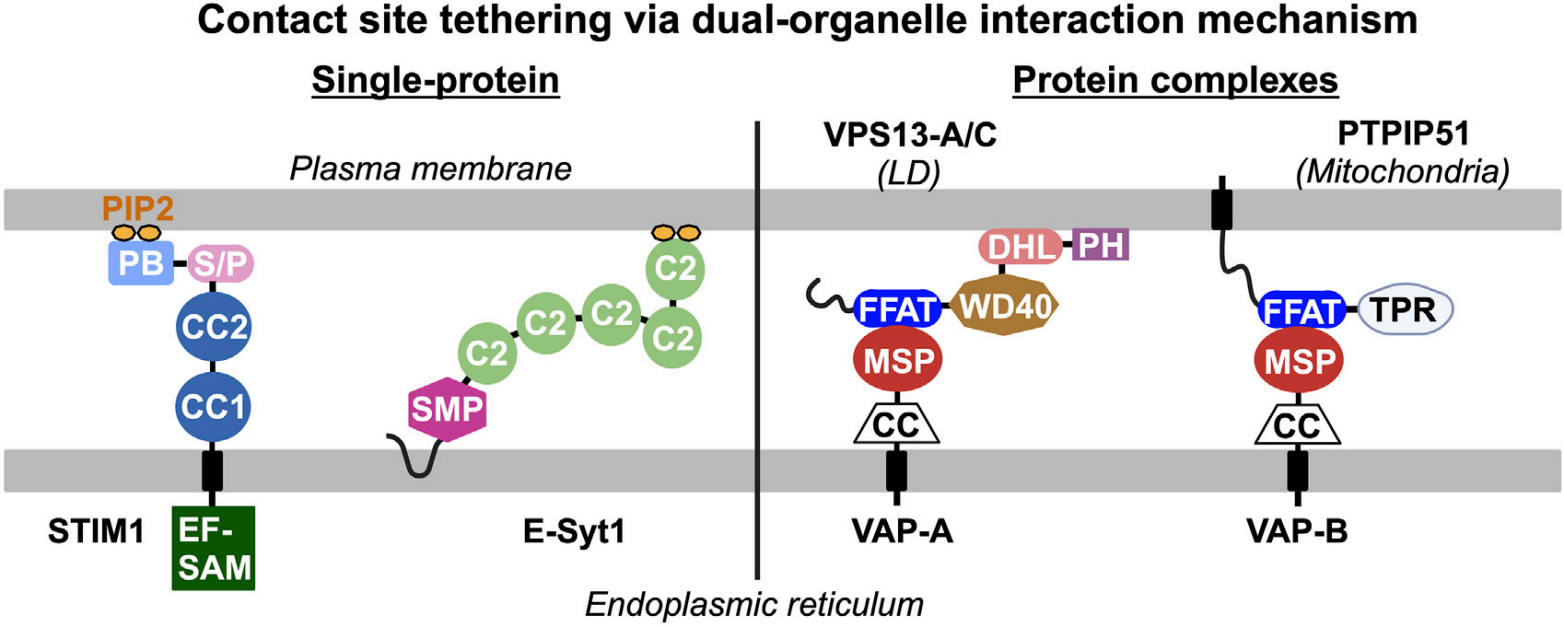
Endogenous tethers and/or tethering complexes at contact sites interact with two heterologous organelles simultaneously. The dual-organelle interaction mechanism is the basis of the design principle for delivering synthetic reporters to specific organelle contact sites. Single protein tethers are exemplified by STIM1 and E-Syt1 that contain an integral endoplasmic reticulum (ER)-associated domain and a lipid-binding motif/domain that binds to phosphatidylinositol 4,5-biphosphate (PIP2) in the plasma membrane (PM), leading to their distribution to ER-PM contact sites. Tethering complexes require protein-protein interaction between two membrane-associated proteins targeted to heterologous organelles to constitute dual-organelle interaction mechanism. For example, the vesicle-associated membrane protein-associated proteins (VAPs) in the ER interact with various lipid transfer proteins (i.e., VPS13-A/C, PTPIP51) that interact with other organelles through their major sperm protein (MSP) and two phenylalanines (FF) in an acidic tract (FFAT) motif, respectively. PB, polybasic motif; C2, C2 domain; S/P, serine and proline rich region; CC, coiled-coil domain; EF-SAM, EF hand and sterile alpha motif; SMP, synaptotagmin-like mitochondrial lipid-binding protein domain; DHL, DH-like domain; PH, pleckstrin homology domain; and TPR, tetratricopeptide repeat domain.

Endogenous proteins and/or protein complexes localized at contact sites must be capable of interacting with two heterologous organelles simultaneously (Figure 1). For example, stromal interacting protein 1 (STIM1) and extended synaptotagmin 1 (E-Syt1) are integral membrane proteins in the ER that harbor a lipid binding motif/domain interacting with phosphoinositides in the PM, resulting in their distribution to ER-PM contact sites (Liou et al., 2005; Liou et al., 2007; Chang et al., 2013; Giordano et al., 2013). Apart from individual proteins with dual-organelle interaction mechanisms, many protein complexes at contact sites rely on protein-protein interactions between two membrane-associated proteins residing in heterologous organelles. The vesicle-associated membrane protein-associated proteins (VAPs) are ER membrane proteins that bind to many lipid transfer proteins capable of interacting with other organelles, such as PTPIP51 in mitochondria and VPS-13 in LDs (Kumar et al., 2018; Yeshaw et al., 2019; Yeo et al., 2021; Kors et al., 2022). Thus, VAP-lipid transfer protein complexes constitute the dual-organelle interaction mechanism for contact site distribution. This dual-organelle interaction mechanism serves as the design principle for delivering synthetic tools to specific organelle contact sites. To better discuss contact site reporters, we have grouped them into three categories: (i) single-protein systems, (ii) two-component systems with proximity-induced reporter, and (iii) reporters for contact site proteomes (Table 1).

**TABLE 1:** Categories of organelle contact site reporters.

Contact site reporters	Contact sites	References
Single-protein system		
MAPPER	ER-PM	Chang et al. (2013), Hsieh et al. (2017), van Vliet et al. (2017), Lee et al. (2019), Ugrankar et al. (2019), Yu et al. (2019), Ruiz-Lopez et al. (2021), Gong et al. (2024), Siegfried et al. (2024)
iMAPPER	ER-PM	Chang et al. (2018)
LiMETER	ER-PM	Jing et al. (2015)
OptoPBer	ER-PM	He et al. (2017)
Dual-component system with proximity-induced reporter signal		
BiFC via traditional FPs	ER-mito; PX-mito; ER-LD; PM-LD, Golgi-PX; vacuole-mito; ER-vacuole; ER-PX; mito-LD; PX-LD; vacuole-LD; vacuole-PX; mito-PM; PX-PM; ER-PM, vacuole-PM	Doghman-Bouguerra et al. (2016), Granatiero et al. (2016), Cieri et al. (2018), Kakimoto et al. (2018), Shai et al. (2018), Yang et al. (2018), Berenguer-Escuder et al. (2019), Vallese et al. (2020), Castro et al. (2022)
BiFC via splitFAST	ER-LD; mito-LD; PX-LD; ER-mito; PM-mito; ER-PM	Casas et al. (2023), Li et al. (2024)
Dimerization-dependent fluorescent proteins (DDFPs)	ER-mito; mito-LD; lyso-LD; ER-LD; PX-LD; PM-LD; caveolin-LD; ER-P-bodies	Naon et al. (2016), Lee et al. (2020), Nguyen and Voeltz (2022), Xie et al. (2022), Miner et al. (2023)
Fluorescence Resonance Energy Transfer (FRET)	ER-mito; mito-lyso; ER-PM	Csordas et al. (2010), Naon et al. (2016), Wong et al. (2018)
Optogenetic dimerization	ER-mito; mito-lyso; ER-lyso; Golgi-ER	Shi et al. (2018), Benedetti et al. (2020)
Proximity labeling		
Contact-ID (split-BioID)	ER-mito	Kwak et al. (2020)
Split-TurboID	ER-mito	Cho et al. (2020a), Cho et al. (2020b)
CsFiND (split-GFP and TurboID)	ER-mito	Fujimoto et al. (2023)

ER: endoplasmic reticulum; PM: plasma membrane; mito: mitochondria; PX: peroxisome; LD: lipid droplet; lyso: lysosome.

## 4 Visualization of organelle contact sites via fluorescent reporters

### 4.1 Single-protein organelle contact site reporter systems

The design of single-protein contact site reporters is exemplified by the topology of STIM1. Thus, all single-protein reporters contain a signal peptide (SP) and transmembrane (TM) domain for ER targeting. Various types of PM-binding motifs have been used for simultaneous interaction with lipids at the PM. These reporters are membrane-attached peripheral ER (MAPPER), light-inducible membrane-tethered peripheral ER (LiMETER), and OptoPBer.

#### 4.1.1 Membrane-attached peripheral ER (MAPPER)

MAPPER is a green fluorescent protein (GFP)-based reporter for ER-PM contact sites. Specifically, the GFP is inserted in between the SP and TM domain of STIM1, followed by cytosolic linkers and a polybasic (PB) motif from a small GTPase, Rit, that binds to phosphoinositides in the PM (Chang et al., 2013). The Rit PB motif is sufficient for the constitutive localization of MAPPER at ER-PM contact sites. The original use of MAPPER revealed a dynamic gap shortening of ER-PM contact sites during calcium signaling, which further supported the mechanistic interrogation of inter-organelle lipid transfer mediated by Nir2 and E-Syt1 (Chang et al., 2013). This gap shortening at ER-PM contact sites was further validated by cryo-EM tomography (ET) (Fernandez-Busnadiego et al., 2015). Over the past decade, MAPPER has been used to monitor the distribution of ER-PM contact sites in diverse cellular processes such as mitosis (Yu et al., 2019), ER stress regulation (van Vliet et al., 2017), cell migration (Gong et al., 2024; Siegfried et al., 2024), cell-cell junction formation (Bharathan et al., 2023), and the disruption of the actin cytoskeleton (Hsieh et al., 2017). In addition, MAPPER is a generalizable tool across species and has been applied to visualize ER-PM contact sites in *Drosophila* (Ugrankar et al., 2019), and *Arabidopsis* and tobacco plants (Lee et al., 2019; Ruiz-Lopez et al., 2021). An inducible version of MAPPER (iMAPPER) was later developed by replacing the Rit PB with the weaker STIM1 PB and introducing a 2xFKBP oligomerization motif (Chang et al., 2018). iMAPPER only localizes to ER-PM contact sites following chemically induced oligomerization. iMAPPER provided proof-of-concept data showing how the microtubule plus-end binding protein EB1 traps STIM1, restraining STIM1’s access to ER-PM contact sites and preventing ER calcium overload during calcium signaling (Chang et al., 2018).

#### 4.1.2 Light-inducible membrane-tethered peripheral ER (LiMETER) and OptoPBer

Another inducible STIM1-based ER-PM contact site reporter is LiMETER. The ER luminal domain region of LiMETER is similar to MAPPER, while the cytosolic region contains a LOV2 (light oxygen voltage-sensing) domain with Jα helix of oat phototropin 1 upstream of the Rit PB motif (Jing et al., 2015). In the absence of light, the Jα helix binds to the LOV2 domain, enclosing the Rit PB motif. When exposed to blue light, a covalent bond forms between a cysteine residue and the flavin cofactor in LOV2, causing the Jα helix to detach and unfold. As a result, the Rit PB motif is exposed, allowing translocation and binding to phosphoinositides in the PM, thereby localizing to ER-PM contact sites. A newer version of LiMETER, OptoPBer, adapted STIM1’s weaker PB for PM binding to resolve the issue of “leaky” ER-PM distribution of LiMETER prior to the activation of blue light (He et al., 2017). OptoPBER spans the distance of less than 10 nm between the ER and PM; this can be further modified with spacers to increase its span up to ∼30 nm. This modification allowed for the observation of the calcium channel ORAI diffusion into ER-PM contact sites (He et al., 2017).

Overall, these synthetic markers substantially improved the detection of ER-PM contact sites with little perturbations to their architecture or functions. The implementation of the single-protein system is straightforward as all the required components for contact site targeting were designed and expressed from one construct. However, this system relies on a known motif/domain that recognizes organelle-specific lipids, hampering its application when organelle-specific lipid-binding mechanisms are not available.

### 4.2 Two-component systems with proximity-induced reporter

The two-component system often utilizes proximity-induced reporters, such as split fluorescent proteins, to achieve bimolecular fluorescence complementation (BiFC) or enhanced signal at contact sites. Each component contains one organelle interaction mechanism. Most split reporters were originally developed to interrogate protein-protein interactions and subsequently adapted to monitor organelle-organelle interaction. This type of design only requires organelle-targeting motifs/domains; thus, enabling the detection of contact sites without known factors. However, this system requires two constructs, each expressing a cognate half reporter targeted to one organelle. Bicistronic elements such as IRES and 2A peptide are often used to control equal expression of the two cognate half reporters.

#### 4.2.1 BiFC at contact sites

A common BiFC reporter used to monitor the contact sites is the split-GFP. Specifically, the GFP1-10 fragment (amino acid residues 1–214) and GFP11 fragment (amino acid residues 215–230) were targeted to several pairs of organelles in previous studies (Cieri et al., 2018; Kakimoto et al., 2018; Yang et al., 2018; Vallese et al., 2020). These reporters have been used to detect ER-mitochondria contact sites in fibroblasts derived from patients with Parkinson’s disease (Berenguer-Escuder et al., 2019), in cells with a mutation in the gene coding for an NADH dehydrogenase subunit (Granatiero et al., 2016), and in adrenocortical carcinoma cells with elevated expression of FATE1 (a member of the mitochondrial fission factor protein family) (Doghman-Bouguerra et al., 2016). Additionally, the split-Venus fluorescent protein, which is a GFP-derived protein with enhanced brightness, was engineered to identify different organelle contact sites in yeast. High throughput colocalization screens in yeast were performed and identified new contact site proteins. Peroxisome-mitochondria contact site proteins (i.e., Fzo1, Pex34) were found to mediate tethering and potentially enable the transfer of β-oxidation products (Shai et al., 2018). More recently, Castro et al. (2022) found 100 new potential contact site proteins and effectors at PM-LD and Golgi-peroxisome contact sites and characterized the contact site protein Lec1, which may facilitate PM-LD ergosterol transport.

While the BiFC method is generalizable, easy to implement, and offers a good signal-to-noise ratio, traditional split FPs may lead to issues like irreversible complementation and fluorescence leakiness that could result in enhanced contact site formation and high background fluorescence, respectively (Romei and Boxer, 2019; Tashiro et al., 2020; Nakatsu and Tsukiji, 2023). A recently developed reversible split fluorescent reporter, splitFAST, provides a solution to these issues. splitFAST was engineered from a 14-kDa fluorescence-activating and absorption-shifting tag (FAST) that reversibly binds hydroxybenzylidene rhodanine chromophores (Tebo and Gautier, 2019; Rakotoarison et al., 2024). Some groups have recently applied splitFAST as the BiFC reporter for visualizing LD-organelle and ER-mitochondria contact sites (Casas et al., 2023; Li et al., 2024). Though its overall fluorescent signal is weaker than in the traditional split FP system, the splitFAST approach has revealed the dynamic regulation of organelle contact sites during distinct metabolic conditions. By incorporating calcium-sensing modules, the splitFAST system is capable of simultaneously detecting contact sites and measuring the associated calcium signals (Casas et al., 2023). Interestingly, high-affinity splitFAST (∼3 μM kd) could significantly enhance organelle association while low-affinity splitFAST (∼220 μM kd) had minimal effects on organelle distribution and interaction (Li et al., 2024; Rakotoarison et al., 2024). These observations suggest organelle contact sites may be maintained by low-affinity tethering complexes. Thus, a high-affinity reversible split reporter is practically irreversible at contact sites, underlining the importance of choosing a BiFC reporter with an appropriate binding affinity. Unfortunately, no study has directly compared splitFAST with GFP1-10/11 to date.

#### 4.2.2 Dimerization-dependent fluorescent proteins (DDFPs)

DDFPs are non-fluorescent monomers which form fluorescent heterodimers in close proximity and are used to visualize organelle contact sites (Alford et al., 2012a; Alford et al., 2012b). This method was often used to assess whether a particular protein is an ER-mitochondria contact site tether or not (Naon et al., 2016; Nguyen and Voeltz, 2022). DDFP contact site reporters also revealed that an ER-PM tether TMEM24 might be functional as well at ER-mitochondria contact sites (Xie et al., 2022) and demonstrated that ER forms contacts with P-bodies (Lee et al., 2020). Recently, this method was used to develop the Contact-FP toolkit for the visualization of various contact sites with potential use for assessing contact site dynamics during nutritional fluctuations and pharmacological perturbations (Miner et al., 2024).

#### 4.2.3 Fluorescence resonance energy transfer (FRET)

A technique also used in detecting organelle contact sites is FRET, which involves the transfer of energy from a donor fluorophore (fused to an organelle-targeting sequence) to an acceptor fluorophore (fused to another organelle motif). This occurs when the two fluorophores are in close proximity as a result of the juxtaposition of heterologous organelles. The use of this method facilitated the visualization of ER-mitochondria contact sites (Csordas et al., 2010) and confirmed the role of a protein as a tether (Naon et al., 2016), but also defined the untethering role of RAB7 GTP hydrolysis at mitochondria-lysosome contacts (Wong et al., 2018).

#### 4.2.4 Optogenetic dimerization

A light-inducible two-component organelle contact site reporter system based on LOV2 called an improved light-induced dimer (iLID) can be used to visualize contact sites between organelles. This system uses the SsrA peptide incorporated into the Jα helix of the LOV2 domain. When in the dark, SsrA is unable to bind to its partner SspB. However, when exposed to blue light, the Jα helix unfolds, allowing SsrA to bind to SspB (Guntas et al., 2015). In this setup, an outer mitochondrial membrane (OMM)-targeting motif is fused to the iLID (LOV2-Jα helix-SsrA) while an ER-targeting sequence is fused to SspB, with fluorescent reporters Venus and mKate2, respectively (Shi et al., 2018). Moreover, the authors demonstrated that this method can be used to control temporal induction of ER-mitochondria tethering by switching the blue light source on and off. They also visualized the spatial induction of ER-mitochondria contact sites by using localized blue light stimulation within a cell. Another example of an optogenetic tool is enhanced Magnets (eMags) (Benedetti et al., 2020) engineered from the yeast Vivid photoreceptor’s N-terminal Ncap domains with complementary charges necessary for dimerization (Kawano et al., 2015). Upon blue light-induced dimerization, eMags pair fused to an organelle targeting sequence and a fluorescent protein allowed the visualization of contact sites between mitochondria-lysosomes, ER-mitochondria, and ER-lysosomes. Additionally, eMags demonstrated an optogenetic control of phosphatidylinositol-4-phosphate transport at Golgi-ER contact sites.

## 5 Identification of contact site proteome via proximity labeling

Monitoring organelle contact sites through live cell imaging is essential to understanding their dynamic behavior. Equally important is the identification of contact site resident proteins which play important roles in the structure and function of contact sites. Genetically encoded proximity-labeling enzymes, such as APEX2 and TurboID, have become powerful tools to unbiasedly identify proteomes in their vicinity (∼20 nm) (Lam et al., 2015; Branon et al., 2018). Recently developed split proximity enzymes, split-BioID, split-TurboID, and split-APEX2, are ideal for identifying contact site proteome via a bimolecular complementation approach with a dual-organelle interaction mechanism. The split-BioID proximity labeling technique was initially employed to investigate protein-protein interactions (De Munter et al., 2017; Schopp et al., 2017). Later, it was adapted to profile the proteome of organelle contact sites. Specifically, a split-pair system of BioID called contact ID was generated by expressing the split-BioID fragments in the ER and mitochondria (Kwak et al., 2020). The authors identified 115 ER-mitochondria contact site proteins, including the FKBP8, which enhances contacts between organelles. One downside of using BioID-mediated protein labeling is the prolonged duration of biotin incubation, which lasts 16 h for split-BioID.

The issue of prolonged biotin treatments with BioID was addressed by performing directed evolution of the bacterial biotin ligase (BirA), resulting in an enhanced version of biotin ligase, TurboID (Branon et al., 2018). TurboID only requires 10 min for biotin labeling. Derived from TurboID, a split-TurboID was further developed to map the proteomes at ER-mitochondria contact sites, validating both already known and uncharacterized contact site proteins (Cho et al., 2020a; Cho et al., 2020b). Recently, a similar approach was also undertaken in yeast to map ER-mitochondria contact site proteins. In addition to split-TurboID, Fujimoto et al. (2023) established a complementation assay system (CsFiND; Complementation assay using Fusion of split-GFP and TurboID) to enable visualization and proteome profiling of ER-mitochondria contact sites. Another method called ABOLISH (Auxin-induced BiOtin LIgase diminiSHing) was developed to reduce the background endogenous biotinylation of proteins in yeast (Fenech et al., 2023). This method uses Bpl1, the only endogenous yeast biotin ligase, which is fused with an auxin-inducible degron (Nishimura et al., 2009; Morawska and Ulrich, 2013), resulting in the enhanced detection of TurboID-labeled protein interactors. The ABOLISH method facilitated the identification of the Lam5 protein at the ER-mitochondria contact site, influencing mitochondrial respiration via a currently unknown mechanism (Fenech et al., 2024).

A split version of APEX2, fused to the OMM and ER, was developed to enable the visualization of ER-mitochondria contact sites using EM and with the potential for proteomic studies of these contact sites (Han et al., 2019). However, APEX labeling relies on the use of hydrogen peroxide, which is toxic to living cells due to its ability to induce oxidative stress.

## 6 Detection of contact sites using advanced EM and imaging analysis

In addition to molecular toolkits, advanced imaging techniques and analyses further provided insights into the architectural details of contact sites. Recently developed focused ion beam scanning EM (FIB-SEM) provides volumetric information at isotropic, nanometer resolution throughout entire cells (Drobne, 2013; Xu et al., 2017; Xu et al., 2020; Heinrich et al., 2021; Collinson et al., 2023). FIB-SEM has been used to identify irregularly shaped peroxisomes that form contacts with multivesicular bodies, recycling endosomes, ERs, and mitochondria (Hoffman et al., 2020), and revealed that ER-mitochondria contact sites often appear near the base of mitochondrial cristae or at regions of constrictions in the OMM (Obara et al., 2024). Notably, Obara et al. (2024) also used single-molecule live-cell fluorescence imaging, detecting the VAPB protein at ER-mitochondria contact sites within the same study.

FIB-SEM was also used to visualize ER-organelle contact sites in mouse brain, providing anatomical references about inter-organelle communication in neurons (Wu et al., 2017). In addition, the use of FIB-SEM revealed ER-mitochondria contact sites and other subcellular architecture in mouse liver under distinct metabolic conditions (Parlakgul et al., 2022). With the recently developed machine learning-based automated segmentation algorithms that help unbiasedly identify and quantify contact sites in the large FIB-SEM data sets (Muller et al., 2021; Nesic et al., 2024), this approach is expected to revolutionize the contact sites field and beyond. In addition, cryo-ET which preserves samples in their native state in vitrified ice (Young and Villa, 2023; Ching et al., 2024) further revealed architectural details of contact sites in yeast. Particularly, the Tricalbin-mediated ER-PM contact sites form curved regions with bridge-like structure (Collado et al., 2019; Hoffmann et al., 2019) and ER-autophagosome contacts, suggesting a direct lipid transfer from the ER during autophagosome formation (Bieber et al., 2022). Furthermore, cryo-ET resolved the *in situ* ERMES complex structure at ER-mitochondria in yeast (Wozny et al., 2023), providing proof-of-concept data of visualizing native protein complexes at organelle interfaces.

Recently developed imaging analysis pipelines also help identify and quantify contact sites from imaging data. For example, an open-source workflow called MiTER allows automated calculation of ER-mitochondria contact sites from confocal images (Kichuk et al., 2024). MiTER script detects the surface area and volume of organelles and their contact sites, and revealed the regulation of ER-mitochondria contacts in yeast in different metabolic conditions. Another membrane contact site detection algorithm, MCS-DETECT, reconstructs the ER-mitochondria contact sites from 3D super-resolution images (Cardoen et al., 2024). DeepContact, a deep-learning protocol, was developed for high-throughput organelle segmentation and contact site analysis from EM images (Liu et al., 2022). In the near future, the combination of advanced microscopy techniques, automated software pipelines, and machine learning algorithms will lead to more objective and precise quantification of organelle contact sites.

## 7 Perspectives

The concept of inter-organelle communication at contact sites was first introduced in the 1950s. However, it was not until the 1990s and early 2000s that the field of contact sites started to gain traction and expand. Advances in imaging technologies heavily contributed to the expansion of contact site research. The future progress in this field will continue to depend on advanced imaging techniques and tools. Specifically, we envision that the newly developed BiFC reporters, such as splitFAST and split-TurboID, will significantly contribute to gaining mechanistic insights into the dynamic regulation of contact sites. Advanced imaging instruments, including super-resolution microscopy and FIB-SEM, with improved spatial-temporal resolution and sampling size, in conjunction with machine learning-based unbiased analyses, will further elevate our understanding of contact site biology to a holistic level. Overall, the anticipated progress will unravel the roles of organelle contact sites in physiology and pathology, leading to new ideas of therapeutic agents for diseases associated with defective organelle contact sites.
